# Motion-robust proton density fat fraction and ${T_2}^*$ mapping in supraclavicular adipose tissue using radial stack-of-stars imaging

**DOI:** 10.1007/s10334-025-01302-x

**Published:** 2025-11-12

**Authors:** Johannes Raspe, Jonathan Stelter, Philipp Braun, Daniela Junker, Mingming Wu, Dimitrios C. Karampinos

**Affiliations:** 1 Institute for Diagnostic and Interventional Radiology, School of Medicine and Health, TUM University Hospital, Technical University of Munich (TUM), Munich, Germany; 2 Deparment of Radiology, LMU University Hospital, Munich, Germany; 3 Laboratory of Magnetic Resonance Imaging Systems and Methods, École Polytechnique Fédérale de Lausanne (EPFL), Lausanne, Switzerland; 4 CIBM Center for Biomedical Imaging (CIBM), Lausanne, Switzerland

**Keywords:** Brown adipose tissue (BAT), Magnetic resonance imaging (MRI), Motion, Radial k-space trajectory

## Abstract

**Purpose:**

Accurate quantification of proton density fat fraction (PDFF) and ${T_2}^*$ in the supracalvicular (SCV) fossa is critical for studying brown adipose tissue (BAT), but is challenged by respiratory motion-induced $B_0$ fluctuations. This study compares conventional Cartesian imaging to a radial stack-of-stars (SoS) trajectory, with and without retrospective temporal $B_0$ correction, in terms of PDFF and ${T_2}^*$ mapping precision.

**Methods:**

Motion-induced $B_0$ fluctuations and tissue displacement were modeled using a digital anatomical phantom. Both Cartesian and radial SoS trajectories were simulated, with temporal $B_0$ correction, relying on oversampling of the *k*-space center, applied to the radial SoS data. Additionally, repeated in vivo scans were performed in four volunteers using both trajectories. PDFF and ${T_2}^*$ were quantified across repetitions.

**Results:**

Simulations demonstrated smaller PDFF and ${T_2}^*$ errors in radial SoS compared to Cartesian imaging under the influence of simulated motion effects. In the simulations, the mean absolute PDFF error decreased from ${1.07\,\mathrm{\%}}_\textrm{PDFF}$ with Cartesian to ${0.47\,\mathrm{\%}}_\textrm{PDFF}$ with radial SoS, and the ${T_2}^*$ error decreased from 7.50 ms to 3.37 ms. In vivo, radial SoS provided higher repeatability for both parameters compared to Cartesian acquisitions, as measured by the inter-scan coefficient of variation. Retrospective temporal $B_0$ correction further improved the repeatability of ${T_2}^*$ quantification.

**Conclusions:**

Radial SoS imaging improves motion robustness and repeatability of PDFF and ${T_2}^*$ quantification in the SCV fossa compared to Cartesian acquisitions. Incorporating retrospective temporal $B_0$ correction further enhances ${T_2}^*$ reliability and may strengthen the precision of BAT activation studies.

**Supplementary Information:**

The online version contains supplementary material available at 10.1007/s10334-025-01302-x.

## Introduction

**Fig. 1:**
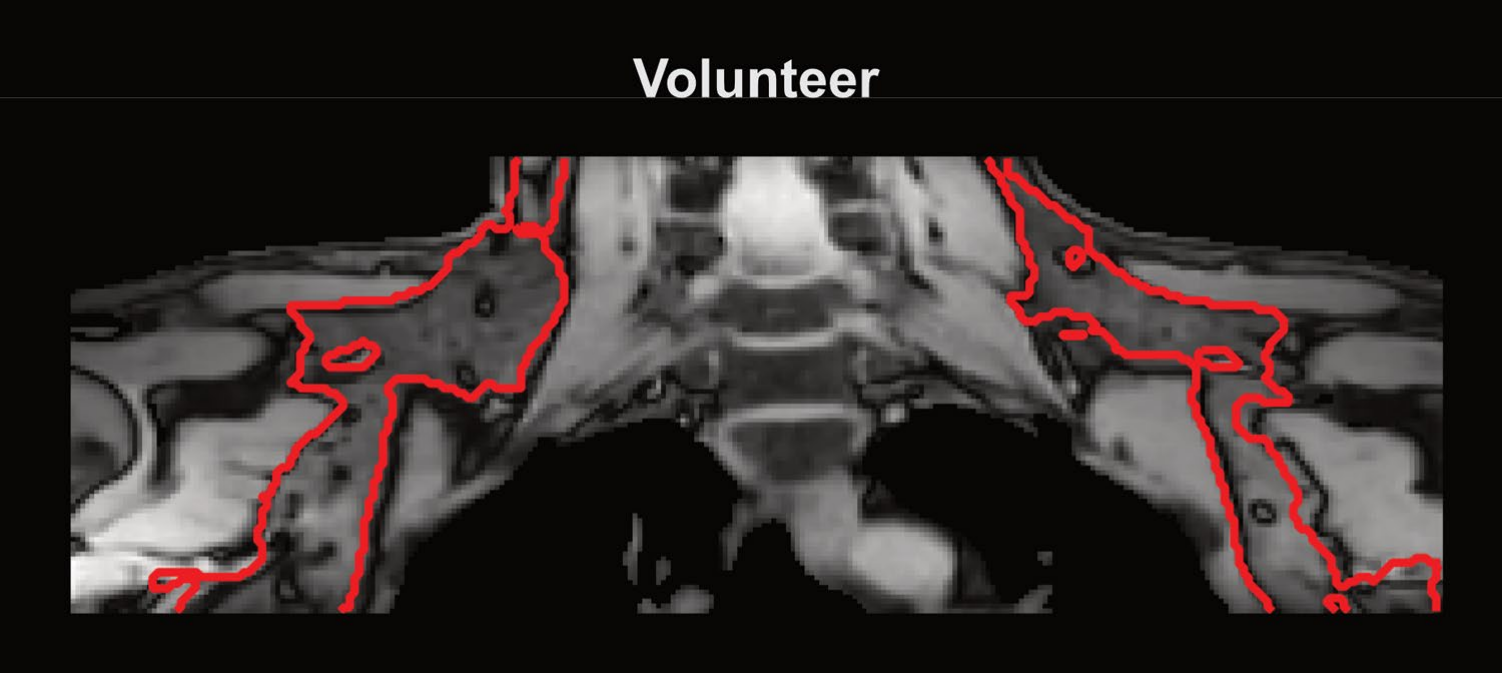
Anatomy of the supraclavicular fossa The underlying image depicts an exemplary coronal slice of a gradient echo image (first echo of a radial scan with scan parameters listed in Table 1) of the upper thorax and neck region. The red segmented region encircles the fat depot of the supraclavicular fossa with the attached axillary fat depot

Magnetic resonance imaging (MRI) is a powerful modality for non-invasive, quantitative tissue characterization. In body imaging, quantitative MRI techniques—in particular proton density fat fraction (PDFF) and ${T_2}^*$ mapping—have proven valuable in assessing tissue composition and function [1–3]. However, many body applications remain technically challenging due to the confounding effect of motion. Physiological motion, such as respiration and cardiac motion, can degrade image quality and introduce inconsistencies in quantitative measurements [4]. Additionally, magnetic susceptibility differences between tissues and at tissue-air interfaces induce static $B_0$ inhomogeneities, which may further fluctuate due to motion, leading to time-dependent $B_0$ fluctuations [5]. While the effects of $B_0$ variations have been studied in the quantitative imaging of different organs, including the brain [6], the breast [7, 8], the prostate [9] and the liver [10], their impact on the supraclavicular (SCV) fossa has not been sufficiently explored. The SCV fossa is a fat-rich region in the lower neck, connected to the cervical and axillary adipose tissue depots. An exemplary in vivo gradient echo image is depicted in Fig. 1.

The SCV fossa is of particular interest because it contains the largest contiguous adipose tissue depot in the adult human body where brown adipose tissue (BAT) can be found [11–13]. BAT imaging has attracted significant interest due to the unique thermogenic function of BAT, its morphological distinction from white adipose tissue (WAT), and its potential role in regulating systemic metabolism. Characterized by smaller lipid droplets than WAT, a high number of mitochondria, and dense vascularization, BAT can be activated by cold exposure or high-caloric meal intake, leading to measureable changes in tissue composition and perfusion [12, 14, 15].

Quantitative MRI—particularly dynamic PDFF and ${T_2}^*$ mapping—has been performed to measure the response of BAT to an activation stimulus in vivo, offering insight into changes of fat content and perfusion during activation [16]. These MRI-based biomarkers offer an attractive, radiation-free alternative to the current gold standard for BAT activation detection, which relies on $^{18}$F-fluorodeoxyglucose ($^{18}$F-FDG) positron emission tomography (PET) with X-ray computed tomography (CT) [17]. Although $^{18}$F-FDG PET/CT can indirectly but reliably detect metabolically active BAT, it cannot differentiate BAT from WAT in the resting state and involves exposure to ionizing radiation. MRI, in contrast, enables radiation-free longitudinal studies, tracking especially changes of PDFF and ${T_2}^*$ over time. However, similar to $^{18}$F-FDG PET/CT, reliable differentiation between BAT and WAT at rest remains challenging using MRI as well [18–20].

The SCV fossa in the neck is an anatomical region close to the lungs with many air-tissue and tissue-tissue interfaces and, thus, many susceptibility differences. Therefore, temporal $B_0$ fluctuations due to respiration can introduce phase changes impacting quantitative MRI, thus reducing repeatability of quantitative MRI measurements. Traditional 3D Cartesian acquisitions have addressed this issue using prospective motion compensation techniques, including breath-holding with multiple breath-holds per acquisition [21–24] or respiratory gating [25–27]. However, these methods are either limited by subject compliance or come with prolonged scan times and result in reduced temporal or spatial resolution for imaging studies dynamically tracking BAT activation. However, dynamic BAT imaging requires high resolution, both temporal and spatial. Since it is necessary to consider a trade-off between temporal and spatial resolution, and scan times are typically above the breath-hold durations, free-breathing acquisitions which are robust to motion and temporal $B_0$ fluctuations are warranted for BAT studies.

*k*-space trajectories that oversample the center of *k*-space are known to reduce the effect of motion on MR images. Radial stack-of-stars (SoS) imaging has emerged as a popular technique for motion-robust abdominal imaging [28]. By repeatedly sampling the *k*-space center, radial trajectories exhibit inherent resilience to motion and are less sensitive to $B_0$ fluctuations. Moreover, the temporal information encoded in the repeatedly sampled *k*-space center enables retrospective corrections to further improve image quality [29].

The purpose of the present work is to characterize the effect of motion on PDFF and ${T_2}^*$ mapping of the SCV fossa and to study the utility of radial SoS imaging for repeatable and precise PDFF and ${T_2}^*$ mapping compared to conventional Cartesian imaging. Furthermore, the impact of retrospective temporal $B_0$ correction to improve repeatability and precision of quantitative measurements relevant to BAT assessment is addressed. Through the performed analysis, the present work seeks to establish a more reliable technique for dynamic fat quantification in the anatomically and physiologically challenging SCV fossa.

## Theory

### Temporal $B_0$ effects

Inhomogeneities in the local main magnetic field $B_0$ are a common source of artifacts in MRI. They may arise from imperfect shimming, magnetic susceptibility differences between tissues and at air-tissue interfaces, or physiological motion. In particular, time-varying $B_0$ inhomogeneities—such as those induced by respiratory or cardiac motion—may lead to dynamic off-resonance effects in gradient echo imaging, described by a spatially and temporally varying frequency shift $\Delta \omega (\textbf{r}, t_j) = \gamma \Delta B_0(\textbf{r}, t_j)$ after a time $t_j = j T_\textrm{shot} + T_\textrm{E}$ of the *j*th shot in the acquisition depending on the local $B_0$ change and the gyromagnetic ratio $\gamma$. This frequency shift even occurs in anatomies not showing any tissue displacements. The resulting phase offset induces errors on the reconstructed images, which depend on the *k*-space sampling trajectory. In Cartesian gradient echo acquisitions, $B_0$ fluctuations cause inconsistent phase offsets between lines of *k*-space, leading to coherent, structured artifacts, such as ghosting, and bias in quantitative maps. In contrast, radial trajectories, such as SoS, repeatedly sample the *k*-space center in each shot. This results in improved robustness to motion, as these inconsistencies are spread across multiple angular projections. Temporal $B_0$ fluctuations affect radial SoS by inducing incoherent artifacts like blurring. The measured gradient echo signal of the radial spoke *j* can be expressed as

1 $$\begin{aligned} S_j(\textbf{k}) = \int {\rho (\textbf{r}) \textrm{e}^{-2\pi \textrm{i}\textbf{k}_j \cdot \textbf{r}} \textrm{e}^{-\textrm{i}\gamma \Delta B_0(\textbf{r}, t_j) T_\textrm{E}}\,\textrm{d}\textbf{r}}, \end{aligned}$$

where $\rho (\textbf{r})$ describes the object magnetization. Variations in the phase offset between spokes lead to inconsistent *k*-space phase, but due to the repeated sampling of the *k*-space center, these effects result primarily in signal loss and image blurring instead of structured artifacts.

### Effect of temporal $B_0$ effects on water-fat separation

Quantitative water-fat separation (WFS) is commonly performed using multi-echo gradient echo acquisition techniques. These rely on a signal model at each voxel of the form

2 $$\begin{aligned} s(T_\textrm{E}) = \left( \rho _\textrm{W} + \rho _\textrm{F} c(T_\textrm{E})\right) \textrm{e}^{\left( 2\pi \textrm{i} \gamma \Delta B_0 - {R_2}^*\right) T_\textrm{E}}, \end{aligned}$$

where the signal $s(T_\textrm{E})$ is described by the complex signals $\rho _\textrm{W}$ and $\rho _\textrm{F}$ of water and fat, the local, time-averaged off-resonance $\Delta B_0$, and a global apparent transverse relaxation rate ${R_2}^*$ [30]. The fat signal is modeled via a multi-peak fat spectrum:

3 $$\begin{aligned} c(T_\textrm{E}) = \sum _{p=1}^{P}{\alpha _p \textrm{e}^{2\pi \textrm{i} f_{\textrm{F},p}T_\textrm{E}}}, \end{aligned}$$

with the number of spectral fat peaks *P*, the relative peak amplitude $\alpha _p$, and the frequency offset $f_{\textrm{F},p}$ of fat peak *p*.

Accurate estimation of $\rho _\textrm{W}$, $\rho _\textrm{F}$, $\Delta B_0$, and ${T_2}^* = \frac{1}{{R_2}^*}$ requires accurate modeling of the signal phase evolution across echo times. However, in the presence of temporal $B_0$ fluctuations, there are additional phase-induced terms that vary during the acquisition of *k*-space and scale with echo time. In Cartesian acquisitions, phase inconsistencies accumulate between *k*-space lines in a semi-periodic manner which leads to frequency modulations of the signal and results in artifacts of increasing severity with longer echo times [25]. Those artifacts in Cartesian acquisitions compromise the multi-echo Dixon fitting process, leading to errors in WFS and parameter estimation. In radial SoS imaging, by contrast, the phase errors cause inter-spoke inconsistencies and are partially averaged due to the oversampling of the *k*-space center. Thus, the phase inconsistencies induce signal loss, which worsens with increasing echo times, but without structured artifacts. However, this can lead to a bias in ${T_2}^*$ [31].

### Correction of temporal $B_0$ effects

To mitigate the effects of temporal $B_0$ fluctuations in radial SoS imaging, a 1D correction approach based on a $B_0$ self-navigator [31] can be applied. This technique leverages the oversampled *k*-space center to estimate phase variations across shots. In each shot *j* of the radial SoS trajectory, spokes are acquired at a fixed angle in the $k_{xy}$-plane for each partition in $k_z$-direction before switching to a new angle and are assumed to be affected by the same phase error. A coil-wise WFS is performed on the central *k*-space signal to estimate the local $B_0$ variation $\delta B_{0,j}(z)$ between two subsequent shots along the partition-encoding direction *z* and the temporal shot dimension. The acquired spoke signal for coil *l* and a given slice $z_0$ after 1D Fourier transform along the partition encoding direction is then corrected by removing the estimated phase error:

4 $$\begin{aligned} S_{j,l}(\textbf{k}, z_0, T_\textrm{E}) = {S_{j,l}}'(\textbf{k}, z_0, T_\textrm{E}) \textrm{e}^{-2\pi \textrm{i}\,\delta B_{0,j}(z_0) T_\textrm{E}}, \end{aligned}$$

where ${S_{j,l}}'$ is the uncorrected spoke signal and $S_{j,l}$ the corrected signal. This slice-wise demodulation realigns the phase across spokes, improving phase consistency and reducing artifacts, but can only correct for spatial variations in the partition encoding direction.

## Methods

### Simulations

**Fig. 2:**
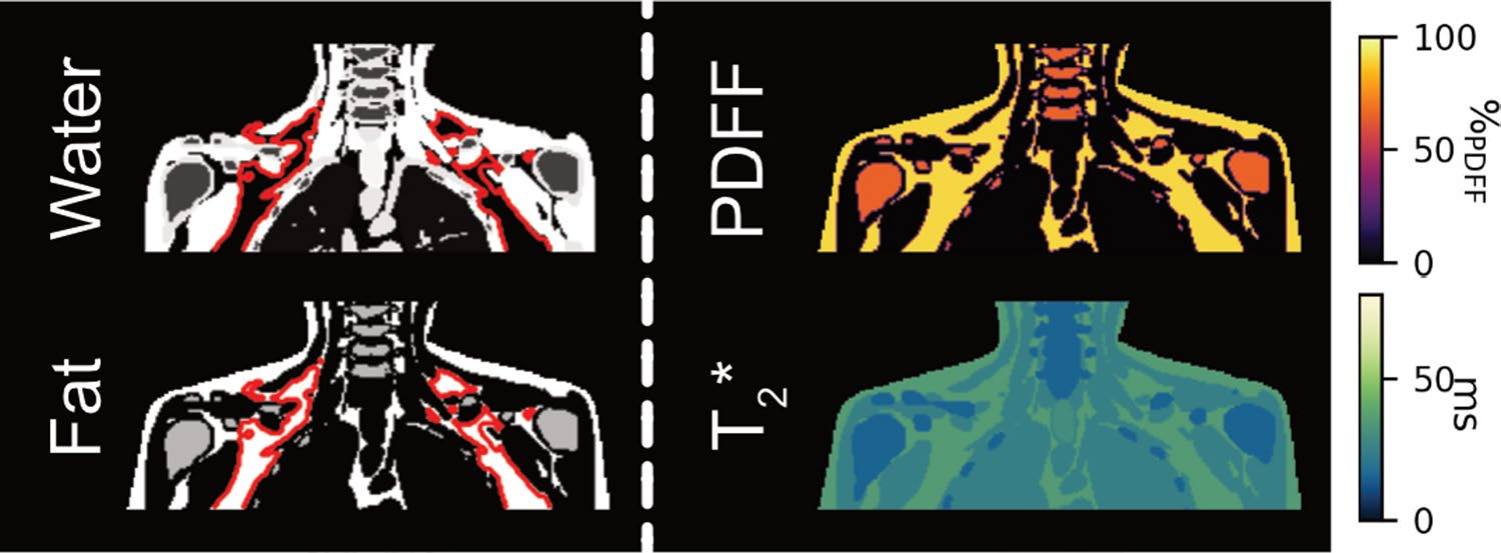
Water-fat separation of the digital phantom. The images on the left depict the simulated water and fat images of the anatomical body phantom from the superior part of the lungs to the neck. The SCV fossa and axillary fat depot are encircled by the red boundaries. The PDFF map on the top right is computed from the fat and water images. The ground truth ${T_2}^*$ map on the bottom right expresses a sharp contrast between low-fat tissues such as muscle and the high-fat containing adipose tissues

Numerical simulations were conducted using the extended cardiac-torso (XCAT) phantom [32], accounting for tissue-specific properties including proton density, fat content, ${T_2}^*$ relaxation, and magnetic susceptibility. The simulated field of view (FOV) covered the upper thorax and neck, including the SCV fossa. Multi-echo gradient echo complex source images were generated based on the signal model in Eq. 2 taking into account a 6-peak fat spectrum [33]. From the complex source images, *k*-space data were forward simulated for both Cartesian and radial SoS trajectories. In addition, water-fat separation was performed to generate ground truth parameter maps. The resulting water and fat images along with the PDFF and ${T_2}^*$ maps are displayed in Fig. 2.

Two types of temporal perturbations were applied to model $B_0$ fluctuations: First, to model time-varying $B_0$ without local tissue displacement through physiological motion, fluctuations were introduced by modifying the original signal model in Eq. 2 with a shot-specific phase offset ${\Delta B_{0,j}}'$ for the *j*th shot as

5 $$\begin{aligned} {s_j}'(T_\textrm{E}) = s_j(T_\textrm{E}) \textrm{e}^{2\pi \textrm{i} \gamma {\Delta B_{0,j}}' T_\textrm{E}}. \end{aligned}$$

The time-dependent ${\Delta B_{0,j}}'$ was modeled as a combination of linear $B_0$ drift and sinusoidal $B_0$ fluctuations according to

6 $$\begin{aligned} {\Delta B_{0,j}}' = \frac{\Delta B_{0,\textrm{drift}}}{N}j + \Delta B_{0,\textrm{fluc}} \sin {\left( 2\pi \frac{T_\textrm{shot}}{T_\textrm{resp}} j\right) }, \end{aligned}$$

with *N* the total number of shots, $\gamma \Delta B_{0,\textrm{drift}} = {5.5\,\mathrm{\text {Hz}}}$ for the Cartesian simulation and $\gamma \Delta B_{0,\textrm{drift}} = {10\,\mathrm{\text {Hz}}}$ for the radial simulation, and $\gamma \Delta B_{0,\textrm{fluc}} = {10\,\mathrm{\text {Hz}}}$ denoting the maximum drift and fluctuation amplitudes, the shot duration $T_\textrm{shot} = {400\,\mathrm{\text {m}\text {s}}}$ for the Cartesian simulation and $T_\textrm{shot} = {470\,\mathrm{\text {m}\text {s}}}$ for the radial simulation, and $T_\textrm{resp} = {5\,\mathrm{\text {s}}}$ the respiratory cycle.

Second, to study the combined effects of motion-induced tissue displacement and $B_0$ fluctuations, 15 different states of tissue displacement within one breathing cycle were simulated. The evolution of signal and $\Delta B_0$ differences is visualized in supplementary Fig. S1. Each state was assigned to shots based on the sequence timing. They were combined to generate a single motion-corrupted *k*-space dataset.

Both Cartesian and radial SoS acquisitions were simulated for 3D multi-echo gradient echo sequences using both 6 echoes and 20 echoes with $T_{\textrm{E},1} = {1.27\,\mathrm{\text {m}\text {s}}}$ and $\Delta T_\textrm{E} = {0.89\,\mathrm{\text {m}\text {s}}}$.

### In vivo measurements

In vivo measurements were performed on a 3 T Ingenia Elition X system (Philips Healthcare, Best, The Netherlands) in four healthy volunteers. First, they were scanned using a 3D multi-echo gradient echo sequence. Each volunteer underwent five scans with a Cartesian readout and five scans with a radial SoS readout without repositioning to assess repeatability. Radial SoS acquisitions employed pseudo golden angle ordering [34] in the $k_{xy}$-plane with 273 spokes per partition, acquiring spokes of one angle in all partitions in $k_z$-direction before switching to a new angle. Second, 300 repetitions of a fast single-echo single-slice coronal gradient echo scan were acquired dynamically with a temporal resolution of 266 ms to map $B_0$ fluctuations at high temporal resolution. Key scan parameters are summarized in Table 1.

**Table 1:** Scan parameters Important scan parameters for the in vivo scans performed in this study are listed below

	2D time-series	3D Cartesian	3D radial
First echo time $T_{\textrm{E},1} \, [{\text {m}\text {s}}]$	5.0	1.12	1.27
Echo spacing $\Delta T_\textrm{E} \, [{\text {m}\text {s}}]$	–	0.9	0.9
Number of echoes $nT_\textrm{E}$	1	6	6
Repetition time $T_\textrm{R} \, [{\text {m}\text {s}}]$	6.0	6.9	7.6
Flip angle $\alpha \; [{ ^{\circ }}]$	5	3	3
shot duration $T_\textrm{shot} \; [{\text {m}\text {s}}]$	266	400	470
Acceleration factor$^{\text {a}}$ *R*	4.5	2	2
Voxel size $V \; [{\hbox {mm}}^{3}]$	(4 $\times$ 4 $\times$ 10)	(2 $\times$ 2 $\times$ 2)	(2 $\times$ 2 $\times$ 2)
Field of view FOV $[{\hbox {mm}}^{3}]$	(300 $\times$ 400 $\times$ 10)	(350 $\times$ 350 $\times$ 144)	(350 $\times$ 350 $\times$ 144)
Slice orientation	Coronal	Axial	Axial
Total scan time $d \; [{\text {min}}]$	1:18	1:12	2:10
	(300 repetitions)		

The single-echo 2D gradient echo was used only to analyze the dynamic $B_0$ changes quantitatively. The 3D sequences were compared in the following sections.

$^{\text {a}}$Acceleration technique: sensitivity encoding (SENSE)

To show repeatability of the radial SoS technique, a phantom containing 15 vials of water-fat emulsions with different fat contents from 0% to 100% (Calimetrix, Madison, WI, USA) was scanned. For that, the radial sequence with the same scan parameters was used. The mean and standard deviation values are shown in supplementary Fig. S2.

### Image reconstruction

Reconstruction from *k*-space data was performed by solving a regularized inverse problem of the form

7 $$\begin{aligned} x = \underset{x'}{\mathrm {arg\,min}}||\mathcal {F}\mathcal {C}x' - y||_2^2 + \lambda ||\textrm{TV}(x')||_1, \end{aligned}$$

where *x* represents the complex-valued multi-echo images, *y* is the complete acquired *k*-space data, $\mathcal {F}$ is the (non-)uniform Fourier operator depending on the trajectory, and $\mathcal {C}$ denotes the coil sensitivity operator. The optimization was solved using an alternating direction method of multipliers (ADMM)-based algorithm with spatial total variation (TV) regularization ($\lambda = 0.5$).

For simulated data, only a single coil was modeled ($\mathcal {C} = \mathcal {I}$), and the Fourier operator $\mathcal {F}$ was implemented as standard fast Fourier transform (FFT) for Cartesian and as non-uniform FFT (NUFFT) for radial trajectories.

For Cartesian in vivo data, vendor reconstructions were used to obtain complex source images directly. For radial in vivo data, off-line reconstruction based on Eq. 7 and NUFFT was performed.

The complex images of the coronal single-echo 2D gradient echo data series for $B_0$ characterization were reconstructed online. The $B_0$ change of voxel *k* was computed according to

8 $$\begin{aligned} \Delta B_{0,k}(t_n) = \frac{\arg {\left( s_k(t_n) {s_k}^*(0)\right) }}{2\pi \gamma T_\textrm{E}}, \end{aligned}$$

with $t_n$ the time of the *n*-th frame within the image data.

### Water-fat separation

WFS was performed offline for both the simulations and the in vivo measurements based on a complex-based water-fat separation approach solving Eq. 2, relying on a multi-resolution graph-cut algorithm [35, 36]. When radial SoS data were used, water-fat separation was performed without any additional temporal $B_0$ correction and with the additional temporal $B_0$ correction based on the 1D Dixon-based self-navigator as described in Sect. 2.3 according to Stelter et al. [31]

### Image analysis

The simulations were compared to the ground truth using the mean absolute error (MAE). Simulations were quantitavely evaluated within an ROI encompassing the SCV and axillary adipose tissue depots as depicted in red on the left in Fig. 1. The MAE was computed for *N* voxels within the ROI according to

9 $$\begin{aligned} MAE = \frac{1}{N} \sum _{i=1}^N{\left| x_i - y_i\right| }, \end{aligned}$$

where $x_i$ and $y_i$ are the *i*th voxel of the ground truth and simulation maps, respectively.

The maps from repeated volunteer scans were compared using an inter-scan coefficient of variation (CV) of an ROI:

10 $$\begin{aligned} CV = \frac{\sigma }{\mu }, \end{aligned}$$

with the standard deviation $\sigma$ and mean $\mu$ over scans. In vivo, circular ROIs with a diameter of 1.6 cm were placed inside the fat depot of the SCV fossa avoiding vessels and muscle tissue.

## Results

**Fig. 3:**
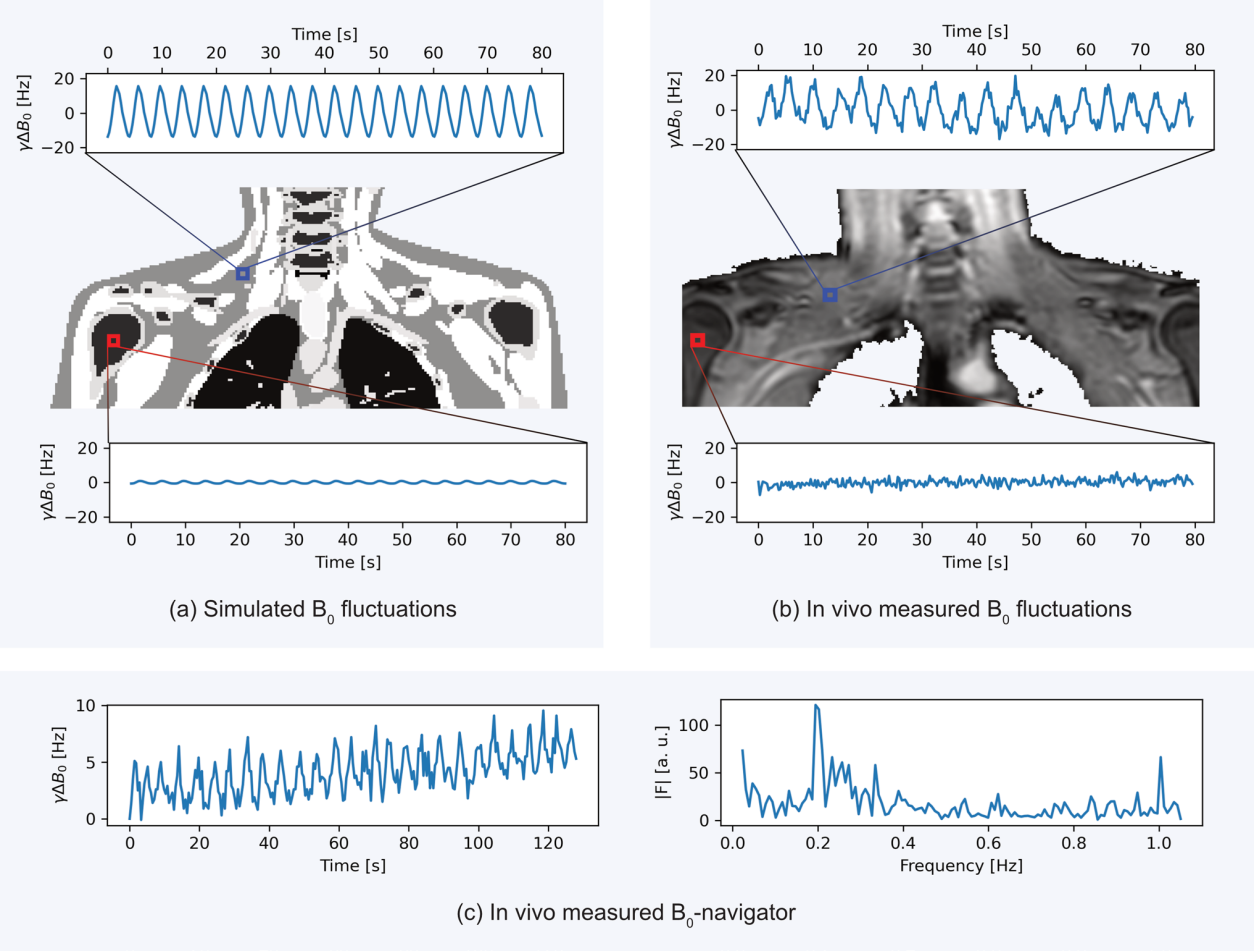
Simulated and measured $B_0$ fluctuations. **a** The image depicts a simulated gradient echo image of the neck region. Two voxels are marked on the coronal slice of the simulated magnitude image. The signal inside the blue voxel placed within the SCV fossa experiences $B_0$ fluctuations in the order of $\pm {15\,\mathrm{\text {Hz}}}$ while the signal fluctuations inside the red voxel in the humerus bone are less pronounced. **b** A similar analysis was performed in vivo with the 2D time-series. The image depicted here is the average magnitude over all 300 frames of the 2D time-series. Again, the blue box marks the SCV voxel and the red box a voxel inside the humerus bone. There are no obvious fluctuations in the bone, but substantial fluctuations in the SCV fossa. **c** Using the $B_0$-navigator with one of the radial SoS scans, global $B_0$ fluctuations as well as a drift over the acquisition time are observable. The Fourier spectrum of the $B_0$-navigator on the right reveals that the main frequency of $B_0$ variations focuses around the breathing frequency of 0.2 Hz

Figure 3 illustrates representative $B_0$ fluctuations in the SCV fossa from both simulations and in vivo measurements. In the simulated data in Fig. 3a, 15 respiratory motion states were modeled over one breathing cycle. A voxel located within the SCV fossa (blue box) exhibited strong $B_0$ fluctuations, with a peak-to-peak range of ${29\,\mathrm{\text {Hz}}}$, while a reference voxel in the humerus bone marked by the red box only showed minor fluctuations of ${1.5\,\mathrm{\text {Hz}}}$.

Similarly, in vivo data acquired with a 2D single-echo scan and processed according to Eq. 8 in Fig. 3b confirmed the $B_0$ fluctuation observation. The SCV fossa voxel again exhibited pronounced, periodic $B_0$ fluctuations with a maximum peak-to-peak deviation of ${41\,\mathrm{\text {Hz}}}$, whereas the bone voxel showed only ${11\,\mathrm{\text {Hz}}}$ deviation. In both cases, $B_0$ in the SCV fossa was more susceptible to respiratory fluctuations.

The presence of global field changes is further supported by the median $B_0$-navigator signal over time from a representative radial SoS scan in Fig. 3c. The navigator shows both a gradual drift and superimposed periodic variations. The frequency spectrum in Fig. 3c confirms that these $B_0$ fluctuations are centered around the expected range of the regular breathing frequency.

### Simulations

**Fig. 4:**
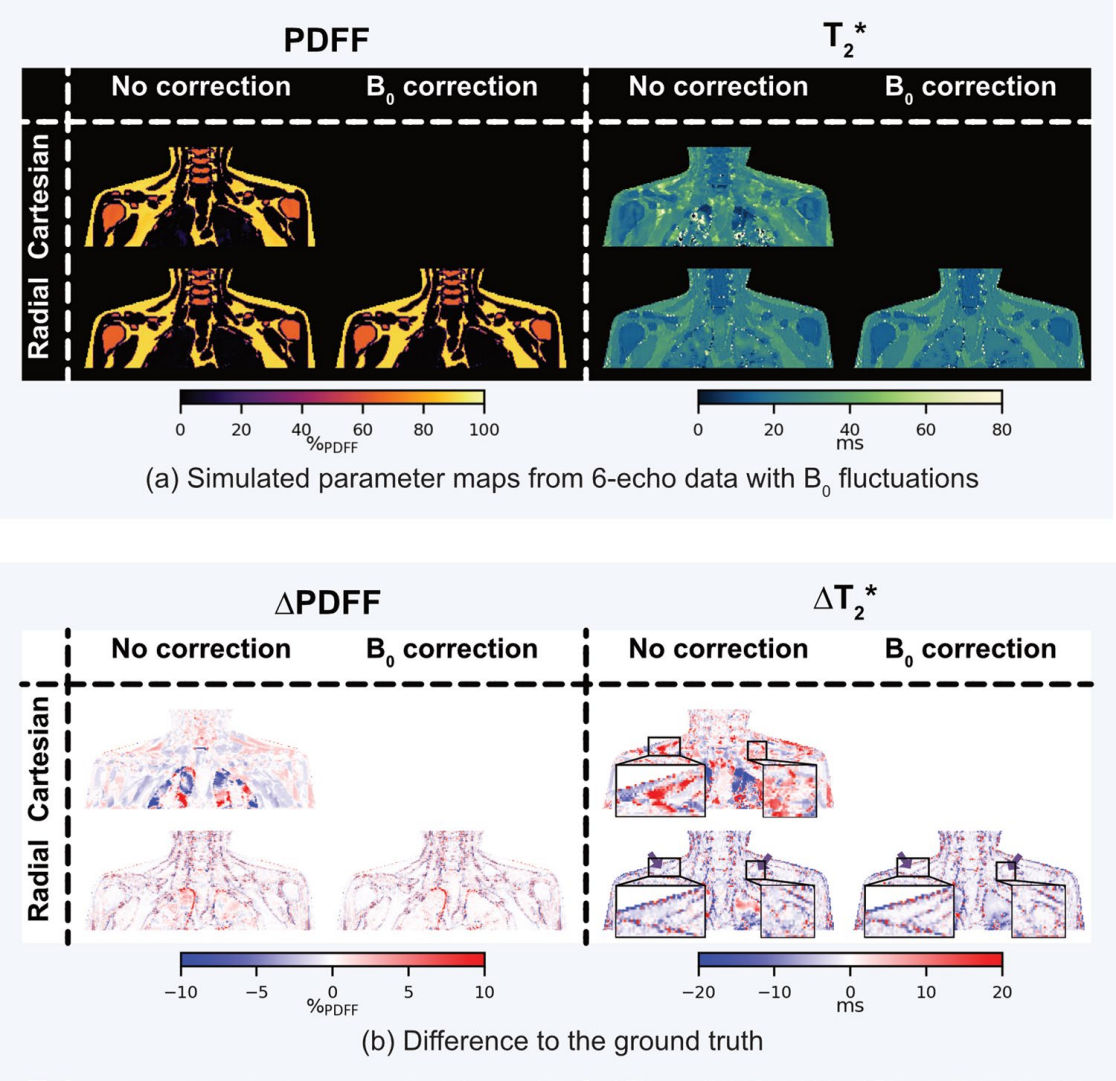
Simulations: only $B_0$ fluctuations without local tissue displacement. **a** The PDFF and ${T_2}^*$ maps from simulated data with additionally simulated $B_0$ fluctuations are shown on the left and right side, respectively. The PDFF maps do not suffer from obvious artifacts with both simulated trajectories. ${T_2}^*$ of the Cartesian simulation has some artifacts superior to the lungs, while the radial SoS does not exhibit visual artifacts. **b** The difference maps of PDFF on the left reveal small errors in the Cartesian PDFF which are modulated with varying positive and negative errors over the FOV. The radial PDFF maps show little difference to the ground truth with small errors at tissue boundaries. The Cartesian ${T_2}^*$ difference map on the right shows similar modulations affecting the whole FOV while the errors in the radial ${T_2}^*$ map are mostly limited to the tissue interfaces. There is a reduction of errors in ${T_2}^*$ applying the temporal $B_0$ correction to the radial SoS reconstruction, including in subcutaneous and SCV adipose tissue (see arrows)

The effect of $B_0$ fluctuations on the simulated PDFF and ${T_2}^*$ maps without local tissue displacement is shown in Fig. 4 for simulations using 6-echo data. The PDFF maps on the left side in Fig. 4a exhibit no obvious artifacts with Cartesian and radial trajectories. The Cartesian ${T_2}^*$ map in the top row on the right exhibits areas of overestimation distributed throughout the image, while there are no primary artifacts visible in the radial SoS ${T_2}^*$ map. The difference maps in Fig. 4b show that the PDFF of radial SoS only has little differences to the ground truth, which are localized primarily at tissue boundaries while slight modulations are observable in the difference map of the Cartesian PDFF. In the Cartesian ${T_2}^*$ map, areas highlighted in red represent the observable areas of ${T_2}^*$ overestimation, which are mixed with areas of underestimation, making it a heterogeneous response to the simulated $B_0$ fluctuations. The ${T_2}^*$ maps from radial SoS maintain a higher level of agreement with the ground truth. The smaller errors in the radial SoS maps compared to the Cartesian maps can also be seen in the error quantification with MAE in the center column of Table 2. The MAE of the radial SoS parameters is lower than in Cartesian for both PDFF and ${T_2}^*$. Employing the temporal $B_0$ correction further reduces the errors, which remain concentrated at tissue boundaries. The reduction of error within the SCV fossa ROI can be observed with a reduced MAE from ${0.47\,\mathrm{\%}}_\textrm{PDFF}$ to ${0.39\,\mathrm{\%}}_\textrm{PDFF}$ in PDFF and from 3.37 ms to 2.79 ms in ${T_2}^*$.

**Table 2:** MAE of simulations listed below are the MAEs between the ground truth and the simulations within the SCV ROI, including only $B_0$ fluctuations without local tissue displacement (center column) and simulations with $B_0$ fluctuations and local tissue displacement (right column)

		$B_0$ fluctuations only	$B_0$ fluctuations and tissue displacement
$MAE_\textrm{PDFF} \; [{\%}_\textrm{PDFF}]$	Cartesian	1.07	5.76
	Radial	0.47	2.42
	Radial corrected	0.39	2.42
$MAE_\mathrm {{T_2}^*} \; [{\text {m}\text {s}}]$	Cartesian	7.50	29.13
	Radial	3.37	19.75
	Radial corrected	2.79	19.85

The errors are consistently smaller with radial SoS compared to Cartesian. $B_0$ correction reduces the errors only in the simulations with $B_0$ fluctuations only.

The parameter maps of $B_0$ fluctuation simulations using 20 echoes are shown in the supplementary Fig. S3. Compared to the 6-echo simulations, PDFF quantification with 20 echoes is not substantially different. The Cartesian ${T_2}^*$ map suffers from fewer artifacts than the 6-echo ${T_2}^*$ while the radial SoS ${T_2}^*$ exhibits higher underestimation but remains close to the ground truth with and without temporal $B_0$ correction.

**Fig. 5:**
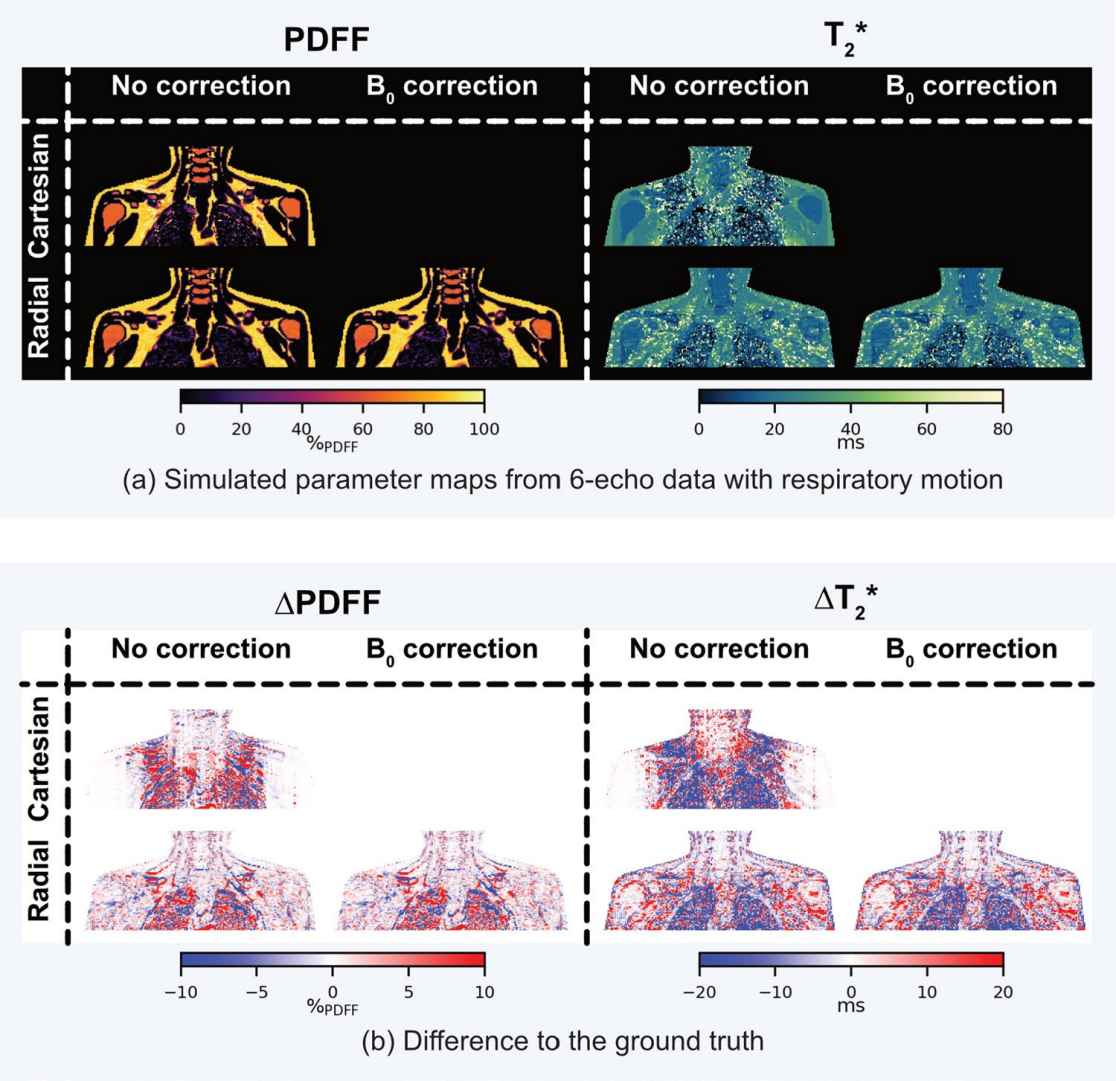
Simulations: respiratory motion including $B_0$ fluctuations and local tissue displacement. **a** The PDFF maps from simulated data with simulated respiratory motion on the left show visible artifacts in the adipose tissue around the lungs in the Cartesian map, while the radial map does not exhibit visible artifacts. The ${T_2}^*$ maps on the right exhibit visual artifacts localized to the areas around the lungs and superior to the lungs in the Cartesian map, while there are artifacts visible over the whole FOV in the radial map, albeit of a smaller extent compared to Cartesian. **b** The PDFF differences to the ground truth on the left show the Cartesian errors affecting the tissues around the lungs including the SCV fossa. Some smaller errors are detectable also in the radial PDFF which are distributed throughout the whole FOV. Similarly, the errors in ${T_2}^*$ on the right are localized superior to the lungs for Cartesian, again including the SCV fossa. There are also large errors in the order of multiple ms in the radial map distributed throughout the FOV. The temporal $B_0$ correction does not visually reduce the errors for both PDFF and ${T_2}^*$

The effect of respiratory motion (taking into account both $B_0$ fluctuations and tissue displacement effects) was simulated and the respective PDFF and ${T_2}^*$ maps for 6-echo data are shown in Fig. 5. Motion affects the Cartesian PDFF map around the lungs and in regions superior to the lungs as shown in Fig. 5a on the left. There are some visible artifacts within the visceral adipose tissue depots with almost no visible artifacts in the radial SoS PDFF map. The ${T_2}^*$ maps are affected by both trajectories. The Cartesian ${T_2}^*$ map again is mostly affected in the areas around and superior to the lungs with areas of vast underestimation mixed with patches of ${T_2}^*$ overestimation. The artifacts are spread out in the radial SoS maps and are of a lower magnitude compared to Cartesian as can be seen by an MAE of 19.75 ms for radial Sos compared to 29.13 ms for Cartesian (see right column of Table 2). Those observations are confirmed by the difference maps in Fig. 5b where the localization of errors to the lung-adjacent areas in the Cartesian case can be seen. The PDFF errors are smaller using the radial SoS trajectory. The ${T_2}^*$ errors are also smaller in magnitude compared to Cartesian, but affect a larger area of the FOV. The temporal $B_0$ correction does not improve the quantitative maps with the respiratory motion simulation. In agreement with the qualitative results, the MAEs in the right column of Table 2 show that the errors are smaller in the radial SoS parameter maps compared to the Cartesian maps but temporal $B_0$ correction does not reduce the errors quantitatively.

The PDFF and ${T_2}^*$ maps of 20-echo data with simulated respiratory motion are shown in the supplementary Fig. S4. For Cartesian, there is a reduction of errors observable compared to the 6-echo simulation for both parameters. In the radial SoS case, PDFF quantification is not improved, while the errors in ${T_2}^*$ are lower compared to the 6-echo simulation.

### In vivo measurements

**Fig. 6:**
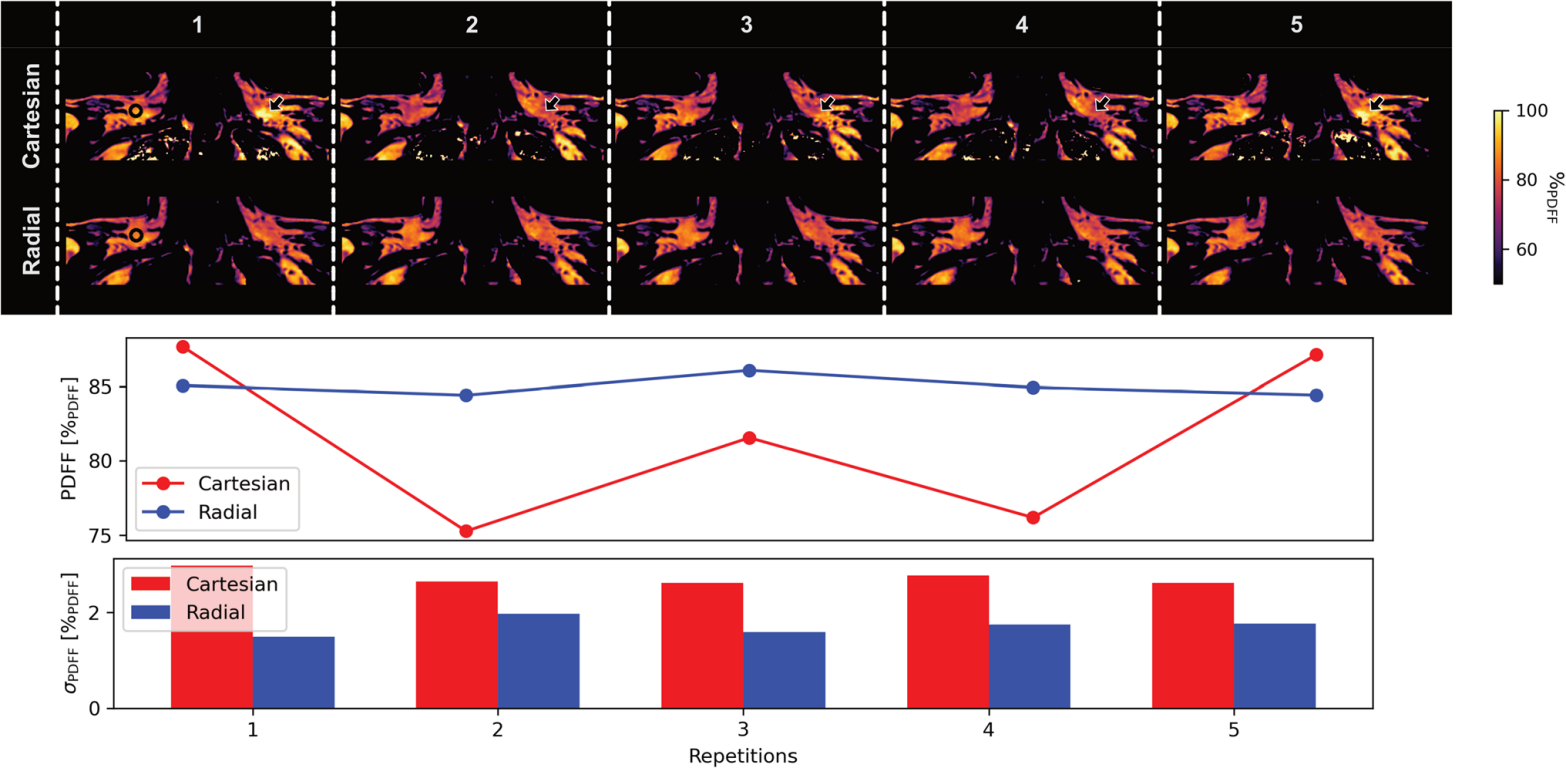
Repeatability of in vivo PDFF mapping in a volunteer. The example Cartesian PDFF maps of five repetitions are shown in the top row and radial SoS maps below. It is worth noting that the range of visualized PDFF values is decreased to highlight the variations in the Cartesian maps. As indicated by the arrows, there are large visual variations of PDFF in the Cartesian case inside the SCV fossa. There are no similar variations observable in the radial SoS maps. The analysis of an ROI indicated by a black circle in the maps on the left shows increased repeatability and reduced intra-ROI standard deviation using radial SoS over Cartesian sampling

Representative PDFF maps from one volunteer are shown in Fig. 6. The maps with Cartesian sampling in the top row exhibit inconsistent overestimation of PDFF in some repetitions, notably, for example, in the left SCV fossa as indicated by the arrows. In contrast, radial SoS maps demonstrate stable and artifact-free maps across repetitions. Quantitative ROI analysis in the right SCV fossa confirms this observation: PDFF values show lower variability with radial sampling, and intra-ROI standard deviation is consistently reduced in the radial SoS PDFF compared to the Cartesian acquisition.

**Fig. 7:**
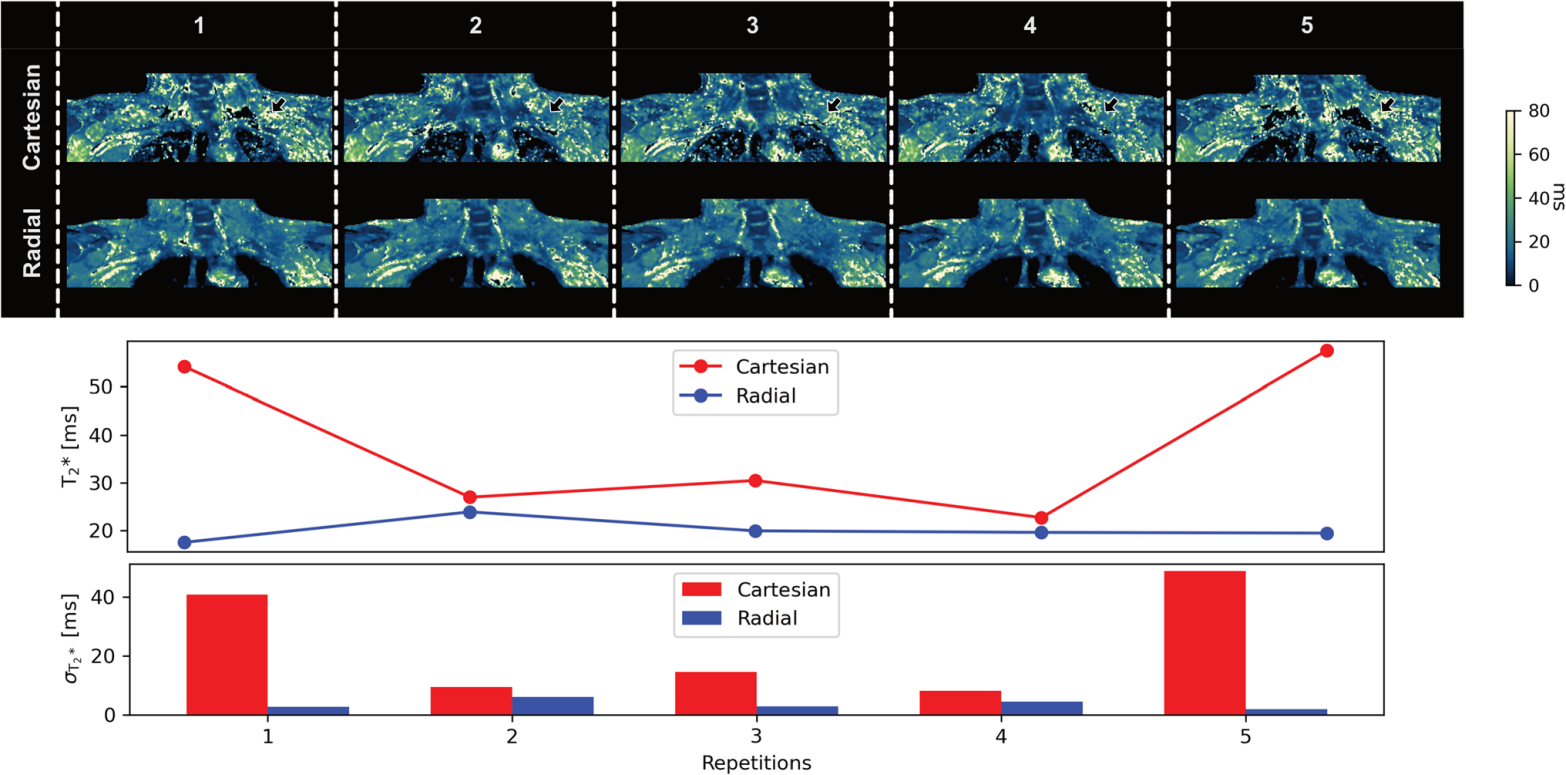
Repeatability of in vivo ${T_2}^*$ mapping in a volunteer. The example slices of the ${T_2}^*$ maps show that Cartesian reconstructions in the top row overestimate ${T_2}^*$ and appear noisy, while radial SoS ${T_2}^*$ maps are more consistent. Mean ${T_2}^*$ variations and intra-ROI standard deviation are lower in the radial ${T_2}^*$ maps compared to Cartesian

The ${T_2}^*$ maps in Fig. 7 show that the maps from the Cartesian reconstructions appear noisier and overestimate ${T_2}^*$, while radial reconstructions are smoother and more consistent across scans. ROI metrics again show reduced inter-scan variability and lower intra-ROI standard deviation with radial SoS scans compared to Cartesian scans.

**Fig. 8:**
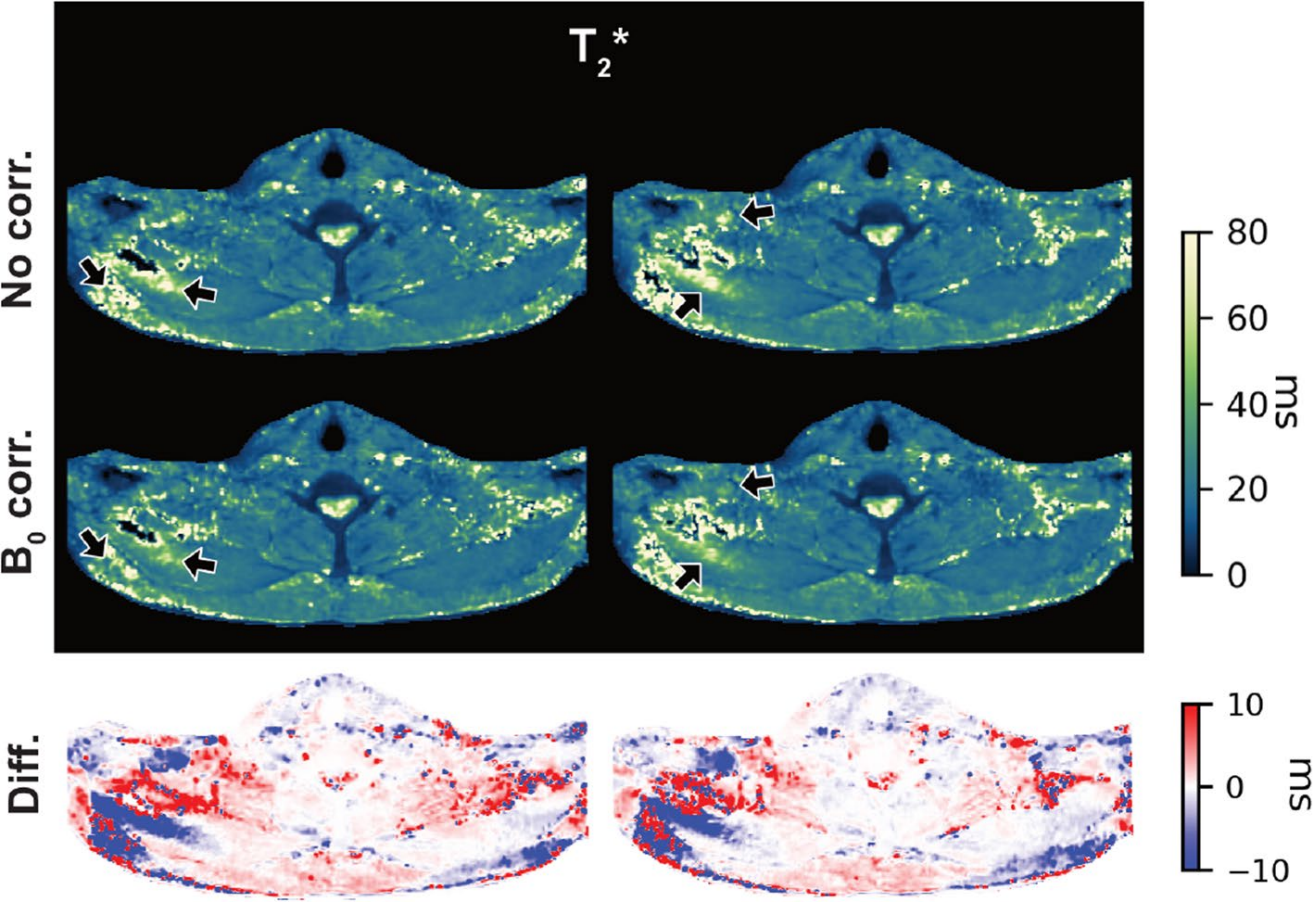
Example ${T_2}^*$ improvement with temporal $B_0$ correction. The two top rows show the uncorrected and corrected ${T_2}^*$ maps, respectively. The arrows point to regions of ${T_2}^*$ overestimation in the uncorrected maps which are reduced after correction. The bottom row shows the difference between the corrected and uncorrected maps

Figure 8 demonstrates the localized effect of temporal $B_0$ correction with the radial SoS acquisitions. The arrows highlight regions of ${T_2}^*$ overestimation in the uncorrected maps, which are reduced after correction. Both muscle and fat-dominant tissues benefit from the temporal $B_0$ correction in the radial SoS acquisitions.

### Group results

**Fig. 9:**
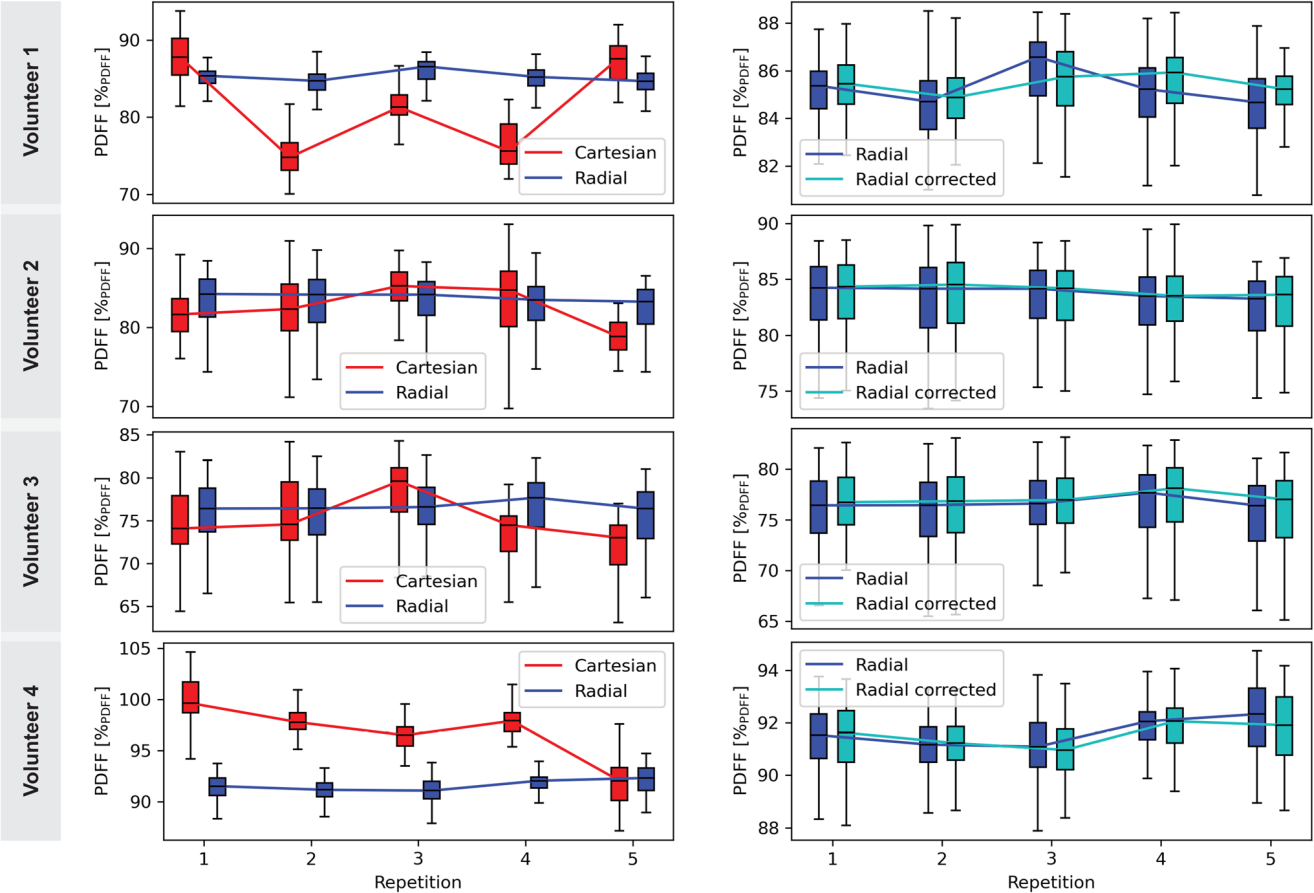
Median PDFF of all volunteers. The left column represents the difference in performance in PDFF mapping with Cartesian compared to radial SoS for all volunteers. The median value is less consistent with a higher variance using the Cartesian acquisitions. The comparison between uncorrected and $B_0$ corrected radial reconstructions in the right column shows that the median gets slightly more consistent after correction

Circular ROIs like for the example volunteer above were evaluated for all volunteers. ROI analysis across all volunteers confirms the trends observed in the single-subject case. As shown in Fig. 9, PDFF values with radial SoS acquisitions are more consistent across repetitions, with lower variance and tighter medians across repetitions compared to Cartesian. Temporal $B_0$ correction has a limited effect on PDFF, though minor improvements are seen in some cases.

**Fig. 10:**
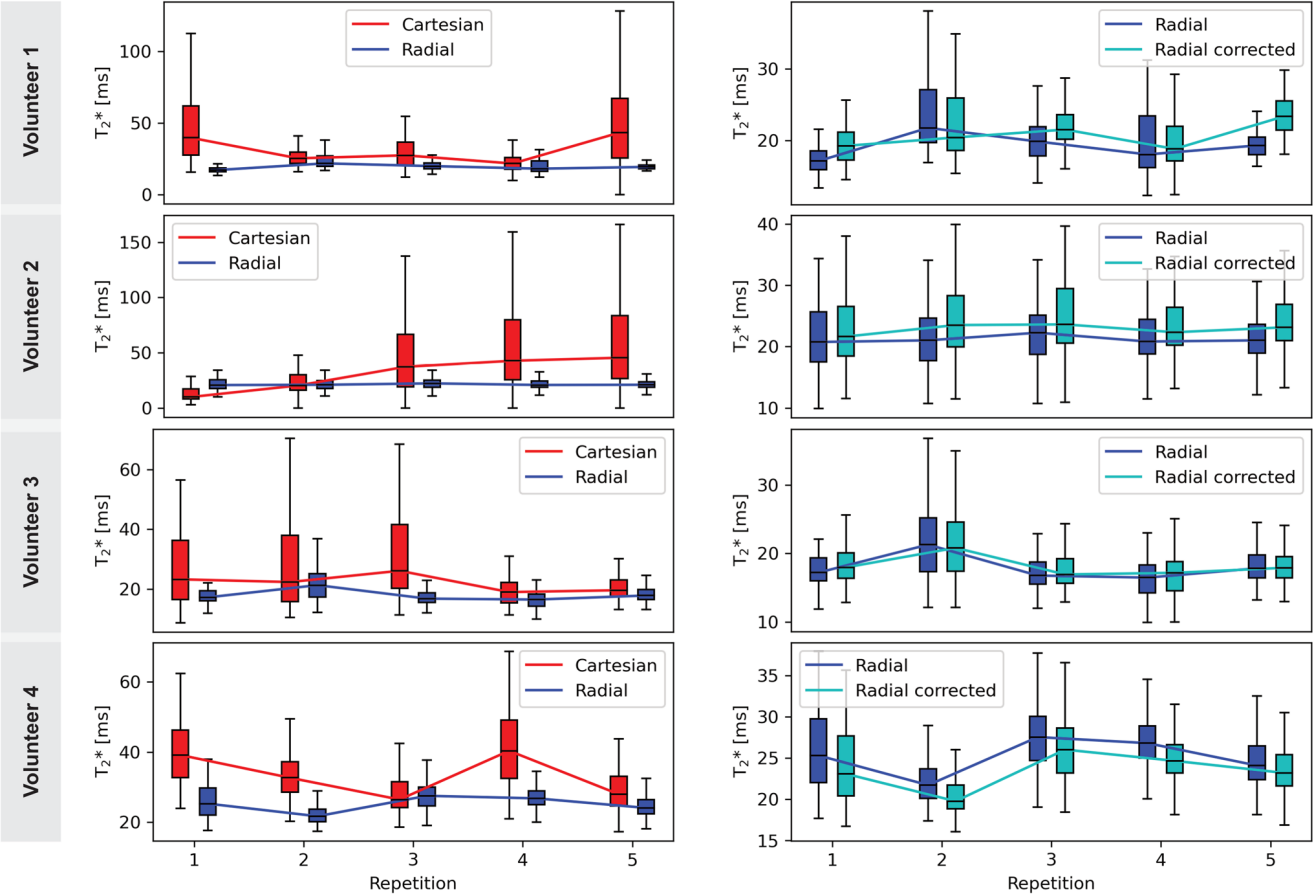
Median ${T_2}^*$ of all volunteers. The left column represents the difference in performance in ${T_2}^*$ mapping for Cartesian compared to radial SoS for all volunteers. The difference between Cartesian and radial SoS in ${T_2}^*$ is even more striking compared to PDFF and radial sampling consistently improves repeatability. The comparison between uncorrected and $B_0$ corrected radial reconstructions in the right column shows that there is a small effect on ${T_2}^*$ quantification when applying the temporal $B_0$ correction

For ${T_2}^*$, differences are more pronounced. The corresponding box plots are shown in Fig. 10. Cartesian acquisitions yield high inter-scan variability, while radial SoS substantially improves repeatability. The repeatability of ${T_2}^*$ with temporal $B_0$ correction is further improved in some cases.

Quantitative summary statistics using the CV across repetitions are listed in Table 3 in the three cases of Cartesian sampling, radial sampling, and radial sampling with temporal $B_0$ correction applied for each volunteer and both parameters PDFF and ${T_2}^*$. It is apparent that the CVs are markedly lower with radial than with Cartesian acquisitions. For ${T_2}^*$ in particular, the CV is reduced by more than ${50\,\mathrm{\%}}$ in all cases. Application of the temporal $B_0$ correction improves CVs in most cases and never worsens PDFF repeatability.

**Table 3:** CV over scans for all volunteers

Volunteer #		V1	V2	V3	V4
$CV_\textrm{PDFF} \; [{\%}]$	Cartesian	6.4	0.8	2.9	2.8
	Radial	0.7	0.4	0.7	0.5
	Radial corrected	0.4	0.3	0.7	0.5
$CV_\mathrm {{T_2}^*} \; [{\%}]$	Cartesian	37.8	32.6	29.1	17.2
	Radial	10.4	8.3	9.6	8.3
	Radial corrected	7.0	6.9	8.1	8.4

The CV across repeated scans for all volunteers and both quantitative parameters is higher with the Cartesian scans compared to the radial SoS scans, showing a consistently improved repeatability in both PDFF and ${T_2}^*$ using radial. Temporal $B_0$ correction further improves repeatability of parameter quantification in most cases.

## Discussion

This study demonstrated that radial SoS imaging offers substantial improvements over conventional Cartesian acquisitions for robust and reproducible PDFF and ${T_2}^*$ quantification in the SCV fossa. Both simulation and in vivo experiments highlight the benefits of radial sampling in mitigating the effects of physiological motion and respiration-induced temporal $B_0$ fluctuations—two major confounders in quantitative MRI of the neck. Radial SoS sampling also enables retrospective correction of motion and $B_0$ effects by leveraging oversampling of the *k*-space center. Furthermore, this study showed that retrospective temporal $B_0$ correction enhances the repeatability of PDFF and ${T_2}^*$ mapping.

Two types of simulations were conducted on a digital anatomical body phantom. In the first simulation, time-varying phase shifts were added to the *k*-space data to simulate $B_0$ fluctuations as an indirect effect of respiratory motion. Cartesian simulations revealed spatially modulated errors in PDFF, and more pronounced in ${T_2}^*$, including in the SCV fossa. In contrast, radial simulations exhibited only minor artifacts, primarily near tissue interfaces. These edges are modeled as sharp transitions in the XCAT simulation. Applying temporal $B_0$ correction further reduced errors in the radial maps—especially for ${T_2}^*$. Increasing the number of echoes from 6 to 20 notably reduced the errors in the Cartesian maps. The second simulation assessed the combined direct and indirect effects of motion on the SCV fossa by averaging different motion states over a breathing cycle. This scenario had a more pronounced impact on PDFF than the first simulation, particularly in the Cartesian simulation. ${T_2}^*$ mapping was affected by motion in both trajectories, with the artifacts stronger and more localized with the Cartesian trajectory. While increasing the number of simulated echoes improved quantification in Cartesian parameter maps, neither a higher number of echoes nor temporal $B_0$ correction substantially benefited radial SoS quantification.

In vivo measurements using 6 echoes supported the simulation findings. Repeated scans of healthy volunteers demonstrated markedly improved repeatability with radial SoS imaging. The repeatability of the radial SoS in a controlled setting was additionally demonstrated in a phantom experiment as the results in supplementary Fig. S2 show. PDFF mapping using Cartesian sequences has already been established and validated previously [37, 38]. However, Cartesian PDFF maps showed occasional regional overestimation and higher inter-scan variability, as reflected by elevated inter-scan CVs, whereas radial SoS parameters were consistently more stable across repetitions. ${T_2}^*$ proved even more sensitive to the choice of sampling trajectory: Cartesian ${T_2}^*$ was characterized by elevated noise and fluctuations in mean and standard deviation within ROIs, while radial SoS imaging yielded smoother and more homogeneous results. Notably, temporal $B_0$ correction reduced inter-scan variability more effectively for ${T_2}^*$ than for PDFF, as measured by inter-scan CV.

In line with previous findings, the simulations confirm that using a larger number of echoes enhances the robustness of Cartesian ${T_2}^*$ estimation to respiratory motion [25, 39]. However, increasing the number of echoes prolongs scan time and may exacerbate sensitivity to other motion artifacts. The simulation and in vivo results indicate that using a radial SoS trajectory instead can provide stable parameter estimation using 6 echoes.

Respiratory and cardiac-induced $B_0$ fluctuations have been reported to influence gradient echo imaging for anatomies located further away from the lungs, including the heart, the cervical spine and the brain [40, 41]. The magnitude of $B_0$ fluctuations was reported to increase closer to the lungs [7]. Investigations on the spinal cord and cerebrospinal fluid within the spinal column revealed more pronounced breathing-induced phase shifts in the lower cervical and upper thoracic spinal cord—regions anatomically close to the SCV fossa [42, 43]. These effects are even more pronounced at higher field strengths, such as 7 T, commonly used in neuro- and spinal cord imaging [41, 42]. Collectively, these observations suggest that the SCV fossa is particularly susceptible to respiration-induced $B_0$ fluctuations and that those fluctuations can significantly impact PDFF and ${T_2}^*$ mapping [25, 31].

To date, three-dimensional Cartesian and radial SoS imaging have not been directly compared with respect to their sensitivity to $B_0$ fluctuations. However, trajectory-specific analyses have highlighted the need for tailored correction strategies. Several correction strategies have been proposed for Cartesian imaging. Following an early *k*-space-based method [40], corrections based on an additional navigator echo were established [44, 45]. Recently, fast single-slice calibration scans have been employed to correct $B_0$ fluctuations in multi-echo gradient echo imaging of the spinal cord [46]. In contrast, radial SoS imaging enables retrospective correction without requiring additional acquisitions of a dedicated navigator or calibration.

The mixed results in improving PDFF and ${T_2}^*$ quantification using radial SoS imaging and retrospective temporal $B_0$ correction likely reflect the substantial inter-individual variability in $B_0$ dynamics, driven by anatomical and physiological factors [41]. For instance, deep breathing was found to have slower but larger $B_0$ fluctuations than shallow breathing [43], leading to varying degrees of $B_0$ correction success. Nonetheless, for prospective studies, employing a sequence that is inherently robust to motion and $B_0$ variations, while enabling retrospective corrections, is advantageous. Additionally, radial SoS trajectories support retrospective motion binning for motion-resolved reconstructions [29]. This is particularly useful when primary motion-induced tissue displacement plays a major role. However, this process discards data from other motion states in the reconstruction, leading to increased undersampling. In the case of the SCV fossa, large tissue displacements are unlikely and reconstructing all available data may provide better results.

These findings have direct implications for in vivo studies of BAT activation, where longitudinal monitoring of PDFF and ${T_2}^*$ has been proposed to assess metabolic activity and responses to external stimuli [16, 47]. Reliable detection of activation-induced changes in BAT requires high precision and reproducibility in quantitative measurements, since PDFF changes typically observed are in the order of 2 %$_\textrm{PDFF}$ to 3 %$_\textrm{PDFF}$ [21–23] and are, therefore, smaller than the observed variations using a free-breathing Cartesian acquisition as shown in Fig. 9. While PDFF remains the most commonly used marker, ${T_2}^*$ provides complementary information related to tissue perfusion and oxygenation, especially during BAT activation, however has not been studied thoroughly.

Previous BAT studies have often overlooked the influence of motion and $B_0$ fluctuations on ${T_2}^*$ measurements, focusing mainly on fat quantification. The findings of the present study underscore that ${T_2}^*$ suffers from lower repeatability under Cartesian sampling, which may partly explain inconsistencies in the literature regarding ${T_2}^*$-based BAT assessment. By adopting a radial SoS trajectory—with optional retrospective temporal $B_0$ correction—both PDFF and ${T_2}^*$ can be more reliably quantified under free-breathing conditions.

This study has several limitations. $B_0$ fluctuations were modeled using a simple combination of linear drift and regular sinusoid according to Eq. 5, which does not account for irregular breathing patterns that potentially degrade ${T_2}^*$ estimation further. Simulated respiratory motion had a larger impact on both PDFF and ${T_2}^*$ quantification than typically observed in vivo, despite the $B_0$ fluctuations being in a comparable range. This discrepancy warrants further investigation. Additionally, simulations were performed without the influence of noise, possibly explaining the observed improvements in ${T_2}^*$ quantification with a higher number of echoes—a trend normally not seen in vivo. However, in vivo scans only included experiments with 6 echoes. Future studies using 20-echo acquisitions may offer further insights. Another limitation is that Cartesian data was reconstructed on the scanner and only WFS was performed offline, while radial SoS data was reconstructed offline to allow for temporal $B_0$ correction. Only 1D $B_0$ correction was implemented in this study, even though the original method [31] also includes a 3D correction which accounts for in-plane temporal $B_0$ fluctuations. Due to considerations of reconstruction time and complexity, only the 1D approach was used here. Exploring the full 3D or other advanced correction strategies in future work could further improve reconstruction robustness.

## Conclusion

In summary, radial SoS imaging enhances the robustness and repeatability of quantitative water-fat MRI in the SCV fossa. It inherently effectively averages motion-related artifacts and provides the flexibility to apply retrospective corrections such as $B_0$ navigators. The resulting improvement in the stability of PDFF and ${T_2}^*$ mapping makes this approach well-suited for longitudinal BAT studies, where small parameter changes need to be tracked reliably over time and across sessions.

## Supplementary Information

Below is the link to the electronic supplementary material.

Supplementary file 1 (pdf 3767 KB)

## Data Availability

Data will be available on reasonable request.
