# Supplementary material for: Motion-robust proton density fat fraction and T2∗ mapping in supraclavicular adipose tissue using radial stack-of-stars imaging
Source: MAGMA. 2025 Nov 12;39(2):237–52. doi: 10.1007/s10334-025-01302-x (PMC13124760; doi:10.1007/s10334-025-01302-x)
Supplement: Supplementary file 1 — (pdf 3767 KB) [file 10334_2025_1302_MOESM1_ESM.pdf]

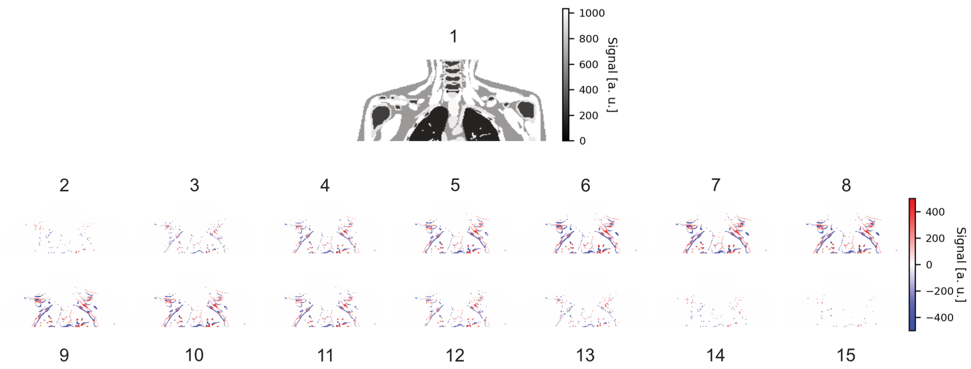

(a) Differences of the first echo magnitude images of the simulated motion states

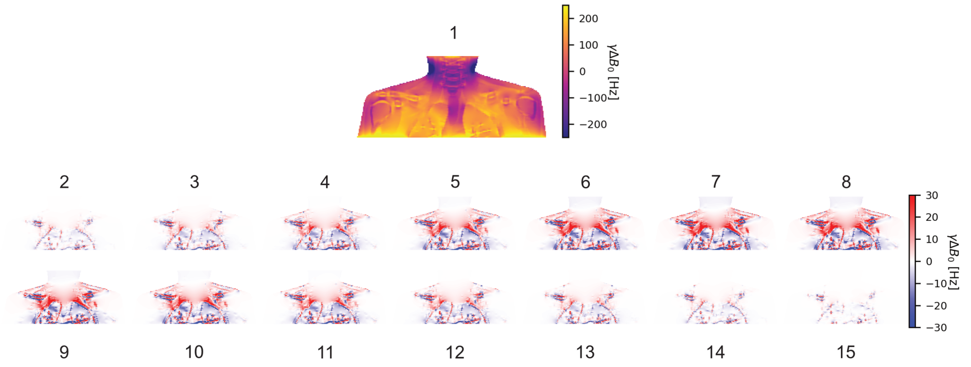

(b) Differences of the ΔB_0 maps of the simulated motion states

Fig. S1 Simulated motion states
(a) Displayed at the top is the first echo image of the first motion state. The remaining images represent the differences between the first motion state and the motion states 2 to 15. Each image depicts the difference of first echo of the n-th motion state to the first motion state. Since the motion states represent one breathing cycle, the magnitudes of the differences are first increasing and decrease for later motion states.
(b) Similar to (a), the top image represents the time-invariant field map of the first motion state. The difference maps visualize how the motion-induced ΔB_0 was simulated over one breathing cycle.

Supplementary information.

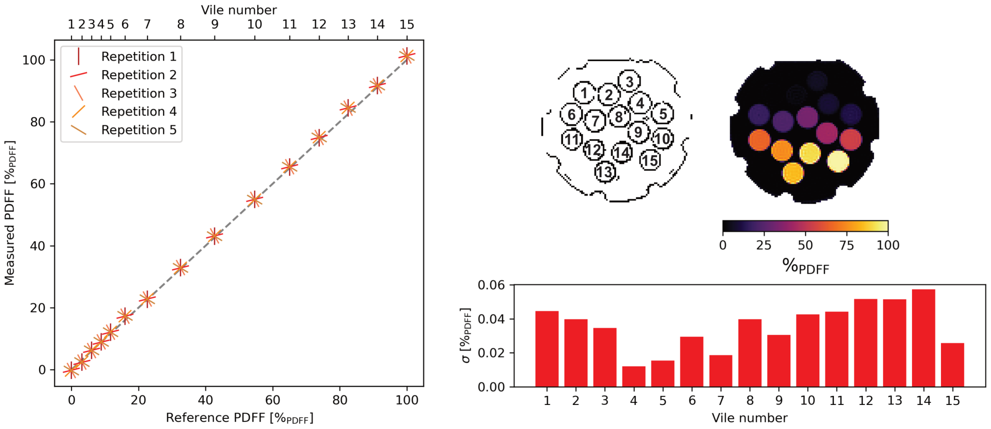

Fig. S2 Repeatability of PDFF in a phantom
The radial sequence used for the in vivo experiments was also applied for five repetitions in the phantom depicted in the top right. It contains 15 vials of different fat content as can be seen in the PDFF map on the right. The mean values in circular ROIs in each vial for all repetitions are plotted on the left and show good agreement with reference values, measured using magnetic resonance spectroscopy. The inter-scan standard deviation per vial in bottom right shows consistently low variations over the full range of PDFF, indicating high repeatability.

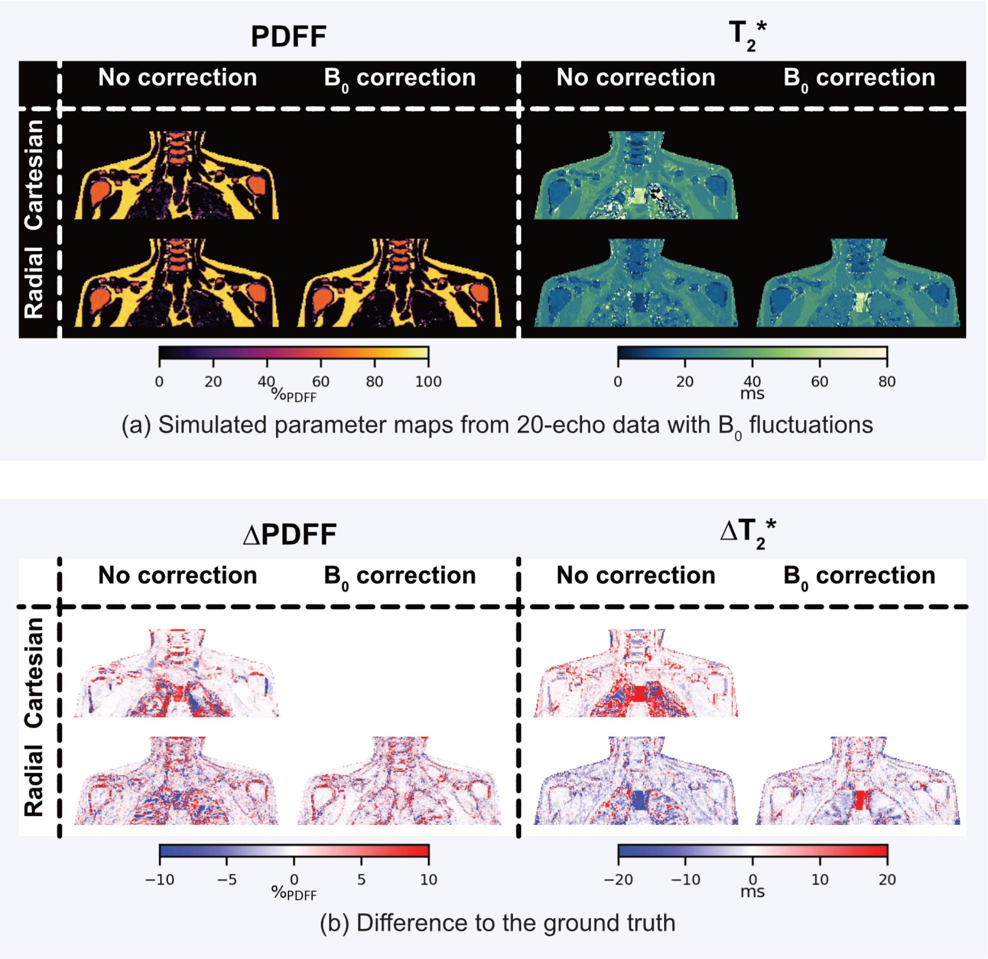

Fig. S3 Simulations: Only B_0 fluctuations without local tissue displacement with 20-echo data

(a) The PDFF maps from simulated 20-echo data with B_0 fluctuations on the left are not affected by visible artifacts. The $T_2{}^*$ maps on the right show little errors, with lower values inside the adipose tissues in the radial SoS map. After temporal B_0 correction, $T_2{}^*$ in adipose tissue appears higher again.

(b) The difference maps for PDFF on the left show that there are little differences to the ground truth map, with slightly higher errors in the radial PDFF map. Those errors are reduced with temporal B_0 correction applied. The $T_2{}^*$ difference maps on the left reveal little errors with Cartesian with some modulations in the adipose tissues including the SCV fossa. The radial SoS map shows $T_2{}^*$ underestimation which is reduced after temporal B_0 correction.

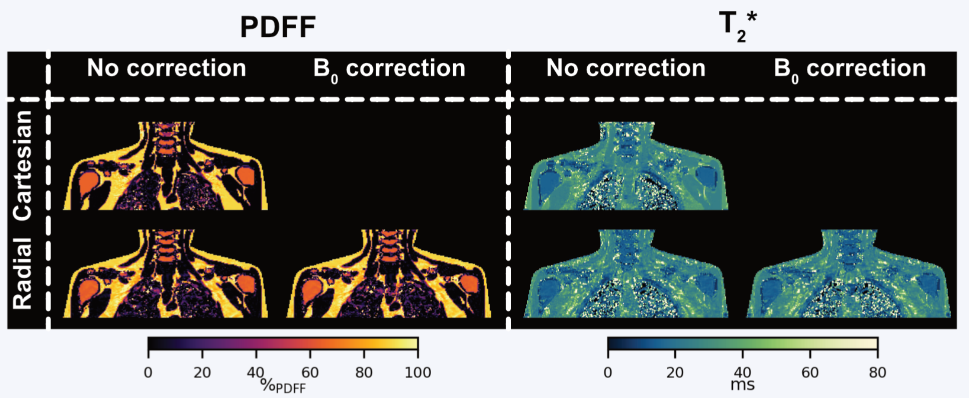

(a) Simulated parameter maps from 20-echo data with respiratory motion

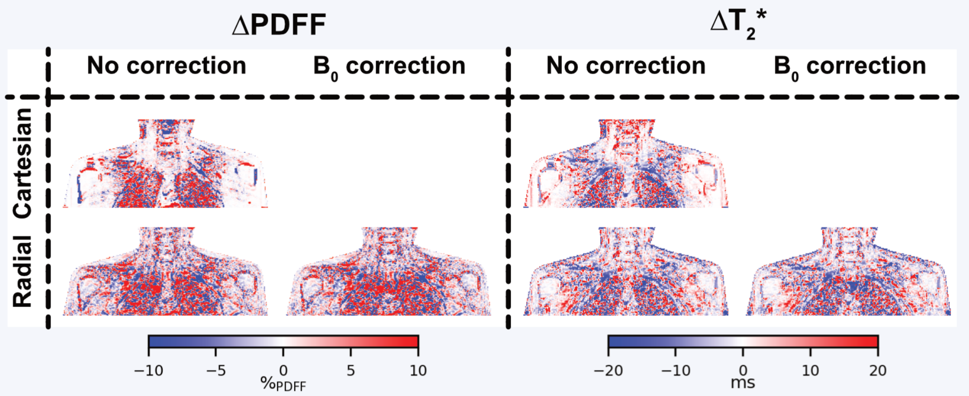

(b) Difference to the ground truth

Fig. S4 Simulations: Respiratory motion including B_0 fluctuations and local tissue displacement with 20-echo data

(a) The PDFF maps from simulated 20-echo data with simulated respiratory motion on the left show visible artifacts in the adipose tissue around the lungs with both trajectories. The T_2* maps on the right exhibit visual artifacts in both the Cartesian and radial SoS maps.

(b) The PDFF differences to the ground truth on the left show the Cartesian errors affecting the tissues close to the lung. The errors are more pronounced in the radial SoS PDFF. The errors in T_2* on the right are localized superior to the lungs, including the SCV fossa, with Cartesian. There are also errors visible in the radial map. The temporal B_0 correction does not visually reduce the errors for both PDFF and T_2*.
